# Supercritical Extract of *Cannabis sativa* Inhibits Lung Metastasis in Colorectal Cancer Cells by Increasing AMPK and MAPKs-Mediated Apoptosis and Cell Cycle Arrest

**DOI:** 10.3390/nu14214548

**Published:** 2022-10-28

**Authors:** Jeong-Geon Mun, Hee Dong Jeon, Dae Hwan Yoon, Yoon Seung Lee, Shin Young Park, Jong-Sik Jin, Noh-Joon Park, Ji-Ye Kee

**Affiliations:** 1 Department of Oriental Pharmacy, College of Pharmacy, Wonkwang-Oriental Medicines Research Institute, Wonkwang University, Iksan 54538, Jeonbuk, Korea; 2 Department of Oriental Medicine Resources, Jeonbuk National University, 79 Gobong-ro, Iksan 54596, Jeonbuk, Korea; 3 Semi Led Co., Ltd., 109 Ballyong-ro, Deokjin-gu, Jeonju-si 54853, Jeollabuk-do, Korea

**Keywords:** *Cannabis sativa*, colorectal cancer, apoptosis, cell cycle arrest, MAPKs, AMPK, metastasis

## Abstract

Colorectal cancer (CRC) is one of the diseases with the highest rates of prevalence and mortality despite therapeutic methods in the world. In particular, there are not enough methods to treat metastasis of CRC cells to distant organs. *Cannabis sativa* Linne (*C. sativa*) is a popular medicinal plant used by humans to treat many diseases. Recently, extracts of *C. sativa* have shown diverse pharmacological effects as a result of choosing different extraction methods. In this study, we performed experiments to confirm the inhibitory effect and related mechanisms of supercritical extract of *C. sativa* on metastatic CRC cells. The effect of SEC on the viability of CRC cell lines, CT26 and HCT116, was determined using CCK reagent. Flow cytometry was performed to confirm whether SEC can promote cell cycle arrest and apoptosis. Additionally, SEC reduced proliferation of CT26 and HCT116 cells without causing toxicity to normal colon cell line CCD-18Co cells. SEC treatment reduced colony formation in both CRC cell lines, promoted G0/G1 phase arrest and apoptosis in CT26 and HCT116 cells through AMPK activation and MAPKs such as ERK, JNK, and p38 inactivation. Moreover, oral administration of SEC decreased pulmonary metastasis of CT26 cells. Our research demonstrates the inhibitory effect of SEC on CRC cell proliferation and metastasis. Thus, SEC might have therapeutic potential for CRC treatment.

## 1. Introduction

Cancer is a multifactorial disease involving a complex interplay of factors, such as genetic cause, exposure to carcinogens, several gene mutations, and inappropriate lifestyle. Colorectal cancer (CRC) is one of a serious disease with progressively increasing incidence and a continuous yearly increase in mortality rate as a result of the aging population and changes in the environment and lifestyle [1]. In South Korea, CRC is a severe disease with a high rate of incidence and mortality [2]. The main treatment methods for CRC patients are resection surgery, radiation therapy, and chemotherapy. However, in ~50% of the total CRC cases, patients do not undergo surgery or radiation. In addition, the 5-year survival rate of advanced-stage patients suffering from metastatic cancer is around 10% [3]. Therefore, it is necessary to discover novel therapeutic agents for CRC patients, especially those with metastatic cancer in distant organs.

Metastasis is a multi-step process that consists of (1) local infiltration of tumor cells into surrounding tissues, (2) intravasation of cancer cells into blood vessels, (3) survival and migration into the circulatory system, and (4) extravasation and invasion in distant organs. During cancer progression, the morphology and phenotype of cancer cells become aggressive. As metastatic cancer is extremely difficult to treat, this is the main reason for cancer-induced mortality [4].

Apoptosis is one of the programmed cell death methods that occur in multicellular organisms. During apoptosis, cell fragments called apoptotic bodies are formed, which are different from cellular necrosis. Since apoptotic bodies are engulfed and removed by phagocytes and do not allow cellular contents to spill out into the surrounding cells, it does not cause damage to other tissues [5]. Therefore, there is a need for a new therapeutic agent that kills cancer cells selectively, excluding the normal cells. In recent years, many studies have provided scientific evidence for inducing apoptosis in cancer cells by regulating the expression of main apoptotic proteins, and using natural products that lead to various antitumor effects [6].

Cannabis plants have been cultivated for medical and industrial use. Cannabis plants have three subspecies of *Sativa*, *Indica*, and *Ruderalis*. However, cultivation and use of cannabis is restricted for common people, but it is conditionally permitted to researchers for scientific purposes [7]. Of the three subspecies of cannabis, *Cannabis sativa* L. (*C. sativa*) has been cultivated since the inception of agriculture, around 10,000 years ago. This plant contains over 400 chemically active compounds. Recently, an increasing number of reports have described the diverse biological activities of cannabis extracts and their compounds [8]. Extracts of *C. sativa* have several pharmacological protective effects such as hepatoprotective, anti-seizure, anti-depressant, and anti-bacterial effects [9,10,11,12]. It has demonstrated the potential to become a novel therapeutic agent for cancer, because it can induce apoptosis in cancer cells, inhibit angiogenesis, and suppress metastatic processes [13,14]. However, there are no reports of *C. sativa* supercritical extracts’ effect on improvement of CRC. Thus, we prepared a supercritical extract of *C. sativa* and confirmed its inhibitory effect on metastatic CRC cells proliferation and pulmonary metastasis in a mouse model.

## 2. Materials and Methods

### 2.1. Extraction of Cannabis Sativa Linne

Supercritical carbon dioxide extract of *C. sativa* (SEC) was prepared using a supercritical fluid extraction system. The strain of *C. sativa* was obtained from the rural development administration of the Republic of Korea. After milling the dried buds (33 g), extract of *C. sativa* was prepared in SFE-2&5LF-2-FMc50 (Waters, Milford, MA, USA). Extraction conditions were 340 bar (pressure), 50 °C (temperature), 2.5 h (time), and 57 g/min (flow rate). The extract was vacuum-evaporated using rotary evaporator N-1000 (EYELA, Tokyo, Japan) and a vacuum aspirator WJ-15 (SIBATA, Tokyo, Japan). For in vitro experiments, the extract was dissolved in DMSO.

### 2.2. Reagents and Antibodies 

A cell counting kit (CCK) for WST assay was purchased from Donginbiotech Co. (Seoul, Korea). Crystal violet solution was purchased from Sigma-Aldrich (St Louis, MO, USA). Anti-phospho-AMPK, phospho-p38, phospho-ERK, phospho-JNK, cyclin D1, CDK4, AMPK, PARP, caspase-3, Bcl-2, Bcl-xL, and Bax antibodies were purchased from Cell Signaling (Danvers, MA, USA). Anti-p38, ERK, JNK, GAPDH, and α-tubulin antibodies were purchased from Santa Cruz Biotechnology (Santa Cruz, CA, USA).

### 2.3. Cell Culture

CCD-18Co (normal human colon cell line) was purchased from American Type Culture Collection (Manassas, VA, USA). Colon 26 (CT26, mouse CRC cell line) and HCT116 (human CRC cell line) were purchased from Korean Cell Line Bank (Seoul, Korea). CCD-18Co cells were cultured in Eagle’s minimum essential medium. CT26 and HCT116 cells were cultured in Dulbecco’s modified Eagle’s medium (DMEM) and RPMI 1640, respectively. The culture medium contained 1% penicillin–streptomycin and the cells were cultured in a CO_2_ incubator (37 °C, 5% CO_2_ atmosphere).

### 2.4. Cell Viability Measurement by WST Assay

Cells were split in a 96-well plate (5 × 10^3^ cells/well) and treated with SEC for 48 h. CCK reagent was mixed with new DMEM and RPMI (1:9) and mixture was added into plate. After 2 h, the absorbance of color change was measured on a microplate reader at 450 nm wavelength.

### 2.5. Colony Formation

CT26 and HCT116 cells were split in a 12-well plate (5 × 10^2^ cells/well) and stabilized overnight. SEC was treated to cells and incubated for 7 days. The medium was removed, and the cells were fixed with 10% formaldehyde. After 15 min, the cells were washed with PBS 3 times. Crystal violet solution was used to stain the colonies for 20 min. After 20 min, the cells were washed with PBS for 3 times, and air-dried and photographed.

### 2.6. Cell Cycle Phase Analysis and Apoptosis Analysis

The percentage of cell cycle phase distribution was determined using the Muse^®^ Cell Cycle Kit (Luminex, Austin, TX, USA). Apoptotic cells were detected with Muse^®^ Annexin V & Dead Cell Assay kit (Luminex, Austin, TX, USA) after SEC treatment. The cells were plated in 6-well plates (5 × 10^5^ cells/well) and treated with SEC for 24 h. After 24 h, the cells were harvested, stained, and analyzed according to the manufacturer’s protocols as previously described [15]. The results were analyzed using Muse cell analyzer (Millipore, Bedford, MA, USA).

### 2.7. Western Blot Analysis

Extraction of total proteins from cells and tissues was conducted using PRO-PREP^TM^ protein extraction solution (iNtRon Biotech, Seoul, Korea) for 45 min. Lysates were mixed with 4× sample buffer and boiled at 95 °C for 5 min. Proteins were separated using SDS-PAGE gel electrophoresis. The immunoblot was blocked using EveryBlot Blocking Buffer (Bio-Rad, Hercules, CA, USA), target molecules were detected with the specific primary and secondary antibodies. After washing with 0.1% TBST, target proteins were visualized using ECL solution in FluorChem M system (ProteinSimple, San Jose, CA, USA).

### 2.8. Establishment of Pulmonary Metastasis Mouse Model

To establish the pulmonary metastasis mouse model, CT26 cells (1 × 10^5^ cells/100 μL of PBS) were injected into the mouse tail vein. D.W or SEC (2.5, 5, and 10 mg/kg) were orally administered to mice once a day until sacrifice. Additionally, 5-Fluorouracil (5-FU, 10 mg/kg) was injected intraperitoneally once every two days as a positive control. After 14 days, the mice were sacrificed, and blood was collected for serological analysis. Nodules in lungs were counted after staining with Bouin’s solution. To analyze specific protein expression, lung tissues were excised and stored in a deep freezer.

### 2.9. Statistical Analyses

The results are expressed as mean standard deviation (SD) of independent experiments, and statistical analysis was performed using Student’s t-test. All statistical analyses were performed using SPSS statistical analysis software version 24 (SPSS Inc., Chicago, IL, USA). Comparisons with *p* < 0.05 were considered to be statistically different.

## 3. Results

### 3.1. Effect of SEC on Viability and Colony Formation of CRC Cells

**Figure 1:**
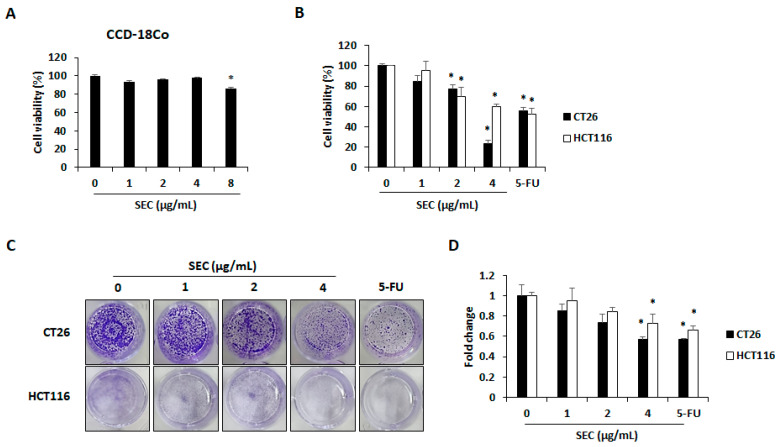
SEC inhibits viability and colony formation of CRC cells. (**A**) Cell viability of SEC-treated CCD-18co cells after 48 h treatment. (**B**) Cell viability in SEC-treated CT26 and HCT116 cells after 48 h of treatment. (**C**) Colony formation in CT26 and HCT116 cells after SEC treatment for 1 week. Images were represented from three independent experiments. (**D**) Counting of colony formation. In this work, 5-fluorouracil (5-FU, 5 μM) was treated as a positive control. The data are representative images and expressed as means ± standard deviation (SD) of experiments. * *p* < 0.05.

To study the selective cytotoxicity of SEC on CRC cells, WST assay was performed on normal colon cell line CCD-18Co cells, and CRC cell lines CT26 and HCT116. At 4 μg/mL concentration, SEC did not cause any cytotoxicity in normal colon cells, whereas 8 μg/mL of SEC significantly decreased cell viability (Figure 1A). Thus, we selected 1, 2, and 4 μg/mL of SEC and observed its effect on CRC cells viability. As shown in Figure 1B, SEC treatment for 48 h reduced the viability of both CRC cell lines. At 4 μg/mL of SEC concentration, the viability of CT26 and HCT116 cells was reduced to 23% and 59%, respectively. Furthermore, SEC inhibited the colony formation of both CRC cell lines in a dose-dependent manner (Figure 1C,D).

### 3.2. Effect of SEC on Cell Cycle Arrest in CRC Cells

**Figure 2:**
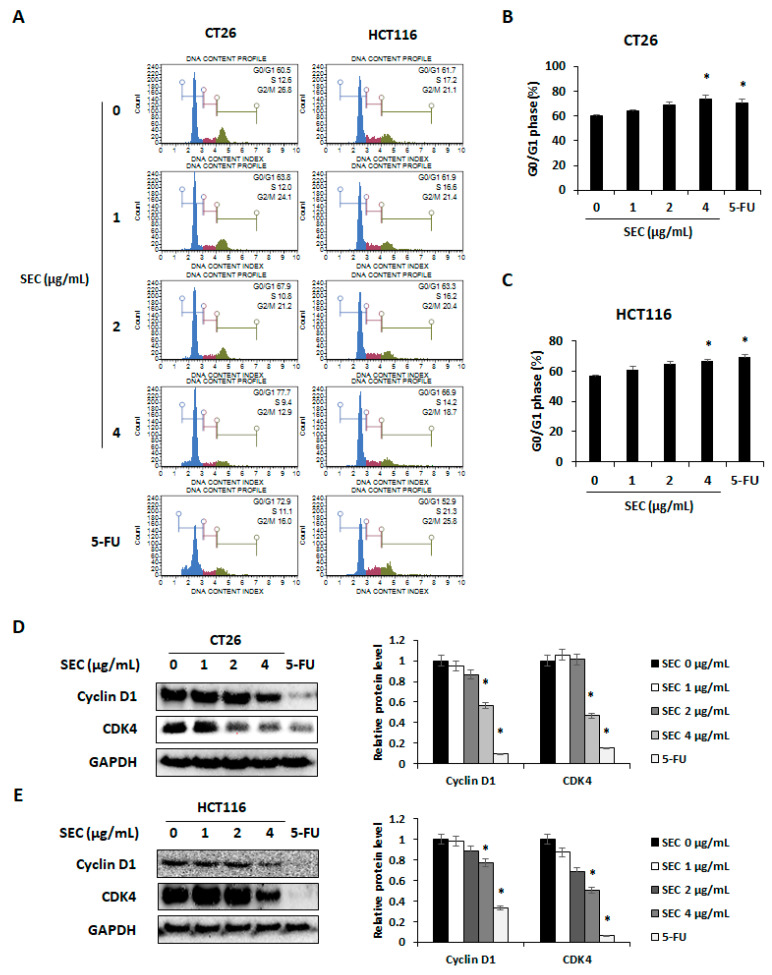
SEC triggers G0/G1 arrest by decreasing cyclin D1/CDK4 expressions in CRC cells. (**A**) Cell cycle phase distribution of SEC-treated CT26 and HCT116 cells was determined by flow cytometry. (**B**,**C**) G0/G1 phase cell population of SEC-treated CT26 (**B**) and HCT116 (**C**) cells. (**D**,**E**) Protein levels of cyclin D1 and CDK4 in CT26 (**D**) and HCT116 (**E**) cells after SEC treatment. In this work, 5-fluorouracil (5-FU, 5 μM) was treated as a positive control. The data are representative images and expressed as means ± standard deviation (SD) of experiments. * *p* < 0.05.

To confirm whether SEC can trigger cell cycle arrest in CRC cells, both CRC cell lines were assessed using flow cytometry. SEC treatment induced G0/G1 phase arrest in CT26 and HCT116 cells (Figure 2A). The percentages of CT26 cells arrested in G0/G1 phase were 63.96%, 68.83%, and 74.23%, in 1, 2, and 4 μg/mL of SEC treatment, respectively. Interestingly, 4 μg/mL of SEC and 5-FU treatment significantly increased the percentage of cells arrested in G0/G1 phase (Figure 2B). In the case of HCT116 cells, the percentage of cells in G0/G1 phase increased (61.16%, 64.66%, and 66.5%), with the increasing concentration of SEC (1, 2, and 4 μg/mL) (Figure 2C). Expression of cyclin D1 and CDK4 is known to be closely related to the progression of G0/G1 phase [16]. Protein levels of cyclin D1 and CDK4 were significantly decreased in CT26 and HCT116 cells by SEC treatment (Figure 2D,E). Therefore, SEC can regulate cyclin D1/CDK4 expression that leads to G0/G1 phase arrest in CRC cells.

### 3.3. SEC Induces Caspase-Mediated Apoptosis in CRC Cells

**Figure 3:**
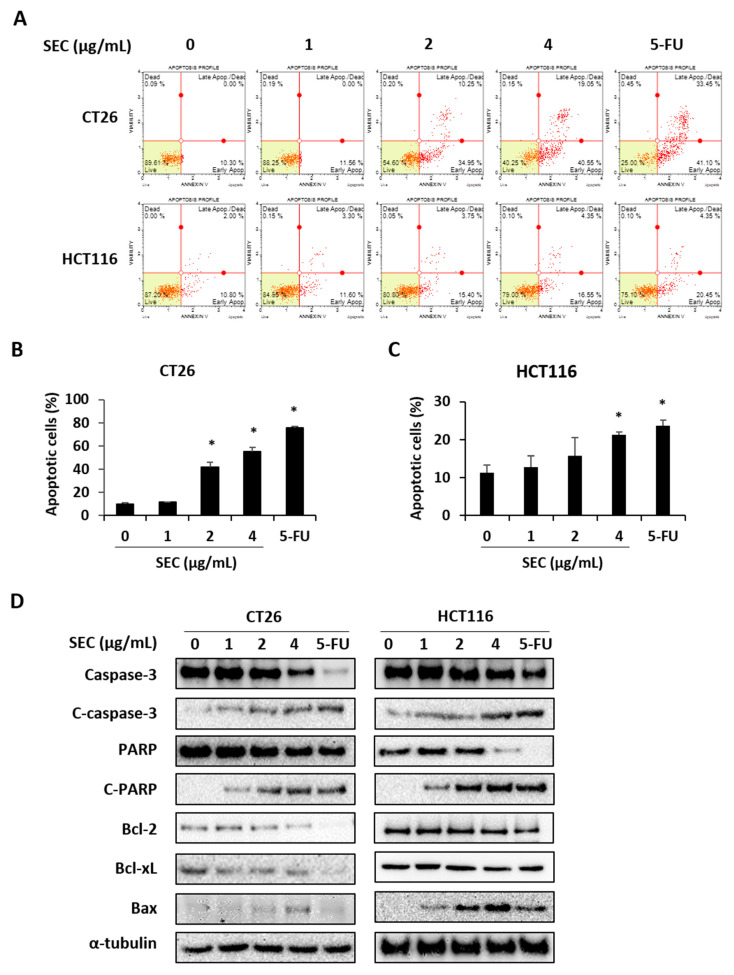
SEC induces apoptosis in CRC cells by regulating apoptosis-associated proteins. (**A**) Annexin V assay data of SEC-treated CT26 and HCT116 cells. (**B**,**C**) Percentages of apoptotic cells in CT26 (**B**) and HCT116 (**C**) cells after SEC treatment. (**D**) Apoptosis-associated proteins including caspase-3, PARP, Bcl-2, Bcl-xL, and Bax in SEC-treated CT26 and HCT116 cells. Data were normalized with α-tubulin. In this work, 5-fluorouracil (5-FU, 5 μM) was used as positive control. The data are representative images and expressed as means ± standard deviation (SD) of experiments. * *p* < 0.05.

To elucidate whether SEC can induce apoptotic cell death in CRC cells, annexin V assay was performed in SEC-treated CT26 and HCT116 cells. The results in Figure 3A show that SEC treatment at 2 and 4 μg/mL concentration increased annexin V-positive cells, which are the apoptotic cells, in both CRC cell lines. At 4 μg/mL SEC treatment, the percentage of apoptotic cells in CT26 cells was 55.06% (Figure 3B). Similarly, 4 μg/mL of SEC also increased percentage of apoptotic cells in HCT116 cells reached up to 21.33% (Figure 3C). To understand the changes in the level of apoptosis-related proteins under SEC treatment in both CRC cells, western blot analysis was performed. SEC increased caspase-3 and PARP cleavages in both CRC cell lines. SEC treatment lowered the expression of anti-apoptotic proteins Bcl-2 and Bcl-xL but upregulated the expression of pro-apoptotic protein Bax in both CRC cells (Figure 3D). These results suggest that apoptosis of CRC cells is caused by SEC treatment via caspase-3 and Bcl-2 family. 

### 3.4. Effect of SEC on Activation of MAPKs and AMPK in CRC Cells

**Figure 4:**
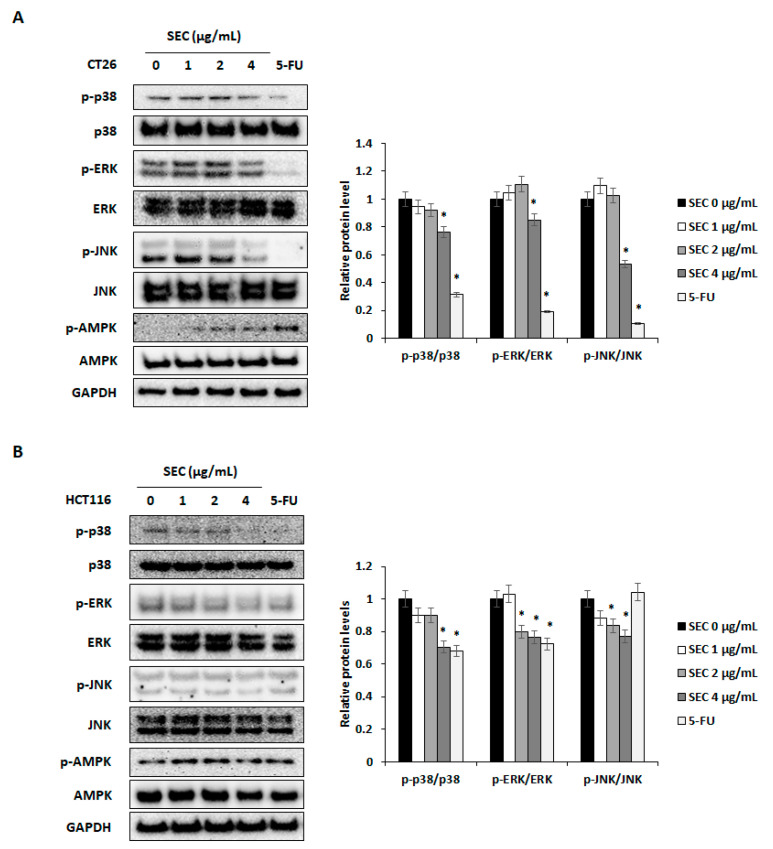
SEC can regulate phosphorylation of ERK, p38, JNK, and AMPK in CRC cells. (**A**,**B**) Western blot results and density of protein bands. Target proteins were visualized from SEC-treated CT26 (**A**) and HCT116 (**B**) cells. SEC and 5-FU (5 μM) was treated to cells (1 × 10^6^ cells/well) for 30 min and cells were harvested to Western blotting. Density values of Western blotting bands were calculated using the ImageJ program. Data were normalized with GAPDH. In this work, 5-fluorouracil (5-FU, 5 μM) was used as a positive control. The data are representative images and expressed as means ± standard deviation (SD) of experiments. * *p* < 0.05.

It has been reported that phosphorylation of MAPKs including p38, ERK, and JNK is necessary for the survival of CRC cells [17]. Additionally, activation of AMPK can lead to apoptosis of CRC cells [18]. To understand the mechanisms involved in SEC-induced cell cycle arrest and apoptotic cell death in CRC cells, target proteins were detected by Western blotting. SEC treatment decreased activation of p38, ERK, and JNK whereas increased activation of AMPK in CT26 cells (Figure 4A). Similarly, SEC inactivated MAPKs and activated AMPK in HCT116 cells (Figure 4B). Thus, SEC can induce apoptosis and cell cycle arrest via MAPKs and AMPK signaling pathway.

### 3.5. Effect of SEC on Pulmonary Metastasis of CRC Cells

**Figure 5:**
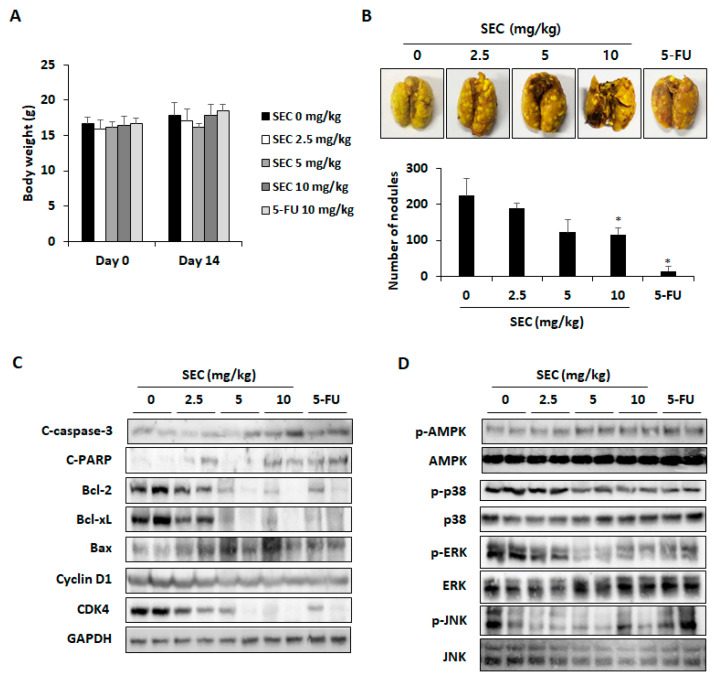
SEC suppressed pulmonary metastasis by inducing apoptosis and cell cycle arrest in CRC cells. (**A**) Body weight of mice during in vivo experiment. (**B**) Representative images of lung tissues and number of nodules in lung tissues. Lungs were stained with Bouin’s solution and nodules were counted. (**C**) Apoptosis-related proteins and cyclin D1/CDK4 protein expression in lung tissues. (**D**) Phosphorylation of AMPK, p38, ERK, and JNK in lung tissues. In this work, 5-fluorouracil (5-FU, 5 μM) was treated as a positive control. The data are representative images and expressed as means ± standard deviation (SD) of experiments. * *p* < 0.05.

To confirm the inhibitory effect of SEC on CRC cell survival, we established lung metastasis mouse model by intravenous injection of CT26 cells. For 14 days, SEC was administrated orally, and 5-FU (10 mg/kg) was administrated intraperitoneally. According to serum analysis results, SEC did not cause hepatotoxicity and nephrotoxicity in mice (Table 1). Additionally, after 14 days, the body weights of SEC-administered mice were similar to those of control group mice (Figure 5A). As shown in Figure 5B, SEC administration inhibited pulmonary metastasis of CT26 cells and 10 mg/kg of SEC decreased number of nodules in lung tissues significantly. To clarify whether SEC can induce apoptosis and cell cycle arrest in CRC cells, the level of apoptosis and cell cycle arrest-associated proteins in lung was detected in lung tissues. SEC promoted activation of caspase-3 and PARP and downregulated Bcl-2 family proteins to induce apoptosis (Figure 5C). Expression of cyclin D1/CDK4, G0/G1 phase arrest-related factors, decreased in the SEC-administered group. Moreover, phosphorylation of AMPK and MAPKs were detected to unravel the molecular mechanisms involved in the inhibitory effect of SEC on pulmonary metastasis in CRC cells. SEC administration increased AMPK activation and decreased phosphorylation of p38, ERK, and JNK (Figure 5D). These results are similar to the in vitro experiment results. Therefore, SEC can block pulmonary metastasis of CRC cells by inducing apoptosis and cell cycle arrest.

## 4. Discussion

To enhance the CRC patient’s quality of life, studies to supplement the drawbacks of existing treatments regimens are being conducted actively. Among the various treatment strategies for CRC, studies are being conducted to find natural products or active compounds that exhibit selective cytotoxicity only for CRC cells [6,19,20]. In addition, the medicinal use of *C. sativa* has recently been the focus by researchers in order to improve many kinds of cancers [21]. Interestingly, supercritical carbon dioxide extraction of *C. sativa* contains more cannabinoids in the extract [22]. A recent study has reported that supercritical extract of *C. sativa* decreases viability of caco-2 cells. IC50 of *C. sativa* supercritical extract is approximately 15 μg/mL. This concentration of *C. sativa* supercritical extract did not exist cytotoxicity to normal human intestinal epithelial cells [23]. However, there is no report explaining the underlying mechanism of the inhibitory effect of *C. sativa* supercritical extract on metastatic CRC. In this study, we prepared extract of *C. sativa* by supercritical extraction method and determined its effect on the viability of metastatic CRC cells after SEC treatment. SEC can inhibit the viability of CRC cells including CT26 and HCT116 cells. 

When DNA damage occurs due to extracellular stimulation, cells pause the cell cycle progression to recover the damage. However, cells undergo cell death such as apoptosis when a cell has been irreparably damaged. In treatment strategy for cancer, there are several types of cancer cell death that have no impact on other cells and tissues. Type І programmed cell death apoptosis is typical of the representative types of cell death without side effects such as chronic inflammation in the microenvironment. Apoptosis is initiated through two pathways which are extrinsic and intrinsic pathway [24]. The death receptor which has been stimulated directly activates caspase-8 in the extrinsic pathway. As a result, it can activate caspase-3 which plays a critical role in the execution phase of apoptotic cell death. In the intrinsic pathway, Bcl-2 family regulates cytochrome c, which can induce cleavage of caspase-9. Activation of caspase-9 can trigger cleavage of caspase-3 which leads to apoptosis of cells. When PARP which is a DNA repair gene is damaged by activated caspase-3, it leads to apoptosis through impaired DNA repair function [25]. It has been also reported that *Cannabis sativa* ethanol extract can induce apoptosis and G1 phase cell cycle arrest through accumulation of intracellular superoxide ions and regulation of mitochondrial membrane potential in human pancreatic cancer cells [26]. In this study, supercritical extract SEC decreased cyclin D1/CDK4 expression, causing G0/G1 phase arrest of CRC cells. Additionally, apoptosis of CRC cells was promoted through regulating caspase-3 and Bcl-2 family by SEC treatment. Therefore, SEC can inhibit CRC cell viability by inducing cell cycle arrest and apoptosis.

It has been reported that cannabis extracts can decrease body weight gain, liver weight, and adipose tissue weight in high-fat/cholesterol diet-induced nonalcoholic fatty liver disease mice model. In the model, during the 6 weeks, 5 mg/kg of cannabis extract was administered in mice by oral administration at every 3 days [27]. Additionally, the effect of cannabis extracts on haloperidol-induced catalepsy and brain oxidative stress was investigated using mouse model. For 18 days, cannabis extract (10 and 20 mg/kg) was administered by subcutaneous injection once a day. Catalepsy was not observed when mice were treated with 20 mg/kg of cannabis extract. At this concentration, cannabis extract decreased malondialdehyde, nitric oxide, and glucose levels in the brain without significant changes in liver malondialdehyde, glutathione, and nitric oxide levels [28]. According to an acute toxicity test, the median lethal dose (LD50) of aqueous cannabis extract is more than 1000 mg/kg and there were no clinical signs of toxicity in the animals. Repeated administration of the aqueous cannabis extract did not show any critical changes in hematological, biochemical, and organ histology parameters. Moreover, cannabis extract (25, 50, and 100 mg/kg) inhibited inflammatory activity in carrageenan-induced edema model, without a spasmolytic effect on airway smooth muscle and central nervous system [29]. In this study, we conducted animal experiments and selected 10 mg/kg of SEC as the highest dose. A maximum 10 mg/kg of SEC was orally administered to mice once a day for 14 days and showed a significant inhibitory effect on pulmonary metastasis of CT26 cells. There were no significant effects on AST, ALT, creatinine, BUN, and body weight. It was observed that administration in the positive control group 5-FU (10 mg/kg) had a more significant inhibitory effect on pulmonary metastasis. However, the factors identified by serological analysis such as AST, ALT, and BUN were slightly increased in the 5-FU administration group compared with SEC administration group. Furthermore, at the end of the experiment, the mice in the 5-FU administration group showed less active behavior than the other groups. Taken together, it is considered that the inhibitory effect of *C. sativa* extract on pulmonary metastasis of CRC cells was demonstrated without significant toxicity in animals.

*C. sativa* is known to contain several active ingredients that are called cannabinoids. Cannabichromene, cannabidiol, cannabigerol, cannabinol, and tetrahydrocannabinol are typical compounds of cannabinoids [30]. In particular, supercritical fluid extraction of *C. sativa* was developed to contain more cannabinoids [31]. Cannabinoids have shown diverse remedial potential against depression, epilepsy, and other effects of clinical relevance [32,33]. Among the various pharmacological effects, several studies have reported an inhibitory effect of cannabinoids on CRC progression. Cannabidiol increases apoptosis in DLD-1 cells through Noxa activation, ROS elevation, and induction of ER stress [34]. Cannabigerol can induce caspase-3-mediated apoptosis of caco-2 cells and inhibit tumor progression in xenograft model and AOM-induced CRC model [35]. Tetrahydrocannabinol suppresses cell survival signaling such as RAS-MAPK and PI3K-AKT signaling pathway. Additionally, this compound induces apoptosis of CRC cells through regulating BAD expression [36]. In this study, SEC can inhibit phosphorylation of MAPKs including p38, ERK, and JNK. Moreover, AMPK was activated in CRC cells by SEC treatment. This result might lead to cell cycle arrest and apoptosis in CRC cells. The supercritical extraction method is a new extraction method capable of extracting many useful ingredients present in medicinal plants. Therefore, it is thought that SEC, the supercritical extract we prepared, induced apoptosis and cell cycle arrest of CRC cells, resulting in cell viability inhibition. Further studies are needed to identify effective compounds in supercritical CO_2_ extract of *C. sativa*.

## Figures and Tables

**Table 1:** Effects of SEC on AST, ALT, creatinine, and BUN serum levels in mice.

	SEC 0 mg/kg	SEC 2.5 mg/kg	SEC 5 mg/kg	SEC 10 mg/kg	5-FU
AST (IU/L)	146.75 ± 71.07	131.75 ± 4.19	133.5 ± 42.79	138.833 ± 21.69	155.66 ± 7.09
ALT (IU/L)	27.25 ± 7.63	37.75 ± 12.14	25.5 ± 5.19	33.4 ± 9.89	40.66 ± 13.05
Creatinine (mg/dL)	0.17 ± 0.03	0.15 ± 0.009	0.14 ± 0.02	0.15 ± 0.03	0.14 ± 0.01
BUN (mg/dL)	19.75 ± 1.70	18.25 ± 1.25	16.5 ± 1.29	20.25 ± 2.36	21.12 ± 6.16

## Data Availability

The data presented in this study are available on request from the corresponding author.
